# Characteristics and prognosis of patients with primary metastatic disease vs. recurrent HER2-negative, hormone receptor-positive advanced breast cancer

**DOI:** 10.1016/j.breast.2025.104412

**Published:** 2025-02-05

**Authors:** Christina B. Walter, Andreas D. Hartkopf, Alexander Hein, Peter A. Fasching, Hans-Christian Kolberg, Peyman Hadji, Hans Tesch, Lothar Häberle, Johannes Ettl, Diana Lüftner, Markus Wallwiener, Volkmar Müller, Matthias W. Beckmann, Laura L. Michel, Erik Belleville, Hanna Huebner, Sabrina Uhrig, Chloë Goossens, Pauline Wimberger, Carsten Hielscher, Julia Meyer, Christoph Mundhenke, Christian Kurbacher, Rachel Wuerstlein, Michael Untch, Wolfgang Janni, Florin-Andrei Taran, Michael P. Lux, Diethelm Wallwiener, Sara Y. Brucker, Andreas Schneeweiss, Tanja N. Fehm, Carlo Fremd

**Affiliations:** a Department of Obstetrics and Gynecology, University of Tübingen, Tübingen, Germany; b Department of Gynecology and Obstetrics, Universitätsklinikum Erlangen, Comprehensive Cancer Center Erlangen-EMN, Friedrich-Alexander-Universität Erlangen-Nürnberg, Erlangen, Germany; c Department of Gynecology and Obstetrics, Marienhospital Bottrop, Bottrop, Germany; d Frankfurt Center for Bone Health, Frankfurt am Main, Germany; e Oncology Practice, Bethanien Hospital, Frankfurt am Main, Germany; f Biostatistics Unit, Department of Gynecology and Obstetrics, Universitätsklinikum Erlangen, Erlangen, Germany; g Department of Obstetrics and Gynecology, Klinikum rechts der Isar, Technical University of Munich, Munich, Germany; h Cancer Center Kempten/Allgäu (CCKA), Klinikum Kempten, Kempten, Germany; i Immanuel Hospital Märkische Schweiz & Immanuel Campus Rüdersdorf, Medical University of Brandenburg Theodor-Fontane, Rüdersdorf bei, Berlin, Germany; j Department of Gynecology, Halle University Hospital, Halle, Germany; k Department of Gynecology, Hamburg-Eppendorf University Medical Center, Hamburg, Germany; l Bavarian Center for Cancer Research (BZKF), Erlangen, Germany; m National Center for Tumor Diseases, Heidelberg University Hospital, German Cancer Research Center (DKFZ), Heidelberg, Germany; n ClinSol GmbH & Co KG, Würzburg, Germany; o Department of Gynecology and Obstetrics, Carl Gustav Carus Faculty of Medicine and University Hospital, TU Dresden, Dresden, Germany; p National Center for Tumor Diseases (NCT), Dresden, Germany; q German Cancer Research Center (DKFZ), Heidelberg, Germany; r Carl Gustav Carus Faculty of Medicine and University Hospital, Technical University of Dresden, Dresden, Germany; s Helmholtz-Zentrum Dresden-Rossendorf (HZDR), Dresden, Germany; t German Cancer Consortium (DKTK), Dresden and German Cancer Research Center (DKFZ), Heidelberg, Germany; u g.SUND Gynäkologie-Onkologisches Zentrum, Stralsund, Germany; v Department of Gynecology and Obstetrics, Klinik Hohe Warte, Bayreuth, Germany; w Department of Gynecology I (Gynecologic Oncology), Gynecologic Center Bonn-Friedensplatz, Bonn, Germany; x Breast Center and CCC Munich, Deptartment of Gynecology and Obstetrics, University Hospital LMU Munich, Munich, Germany; y Department of Gynecology and Obstetrics, Helios Clinics Berlin-Buch, Berlin, Germany; z Department of Gynecology and Obstetrics, Ulm University Hospital, Ulm, Germany; aa Department of Obstetrics and Gynecology, University Medical Center Freiburg, Freiburg, Germany; ab Department of Gynecology and Obstetrics, Frauenklinik St. Louise, Paderborn, St. Josefs-Krankenhaus, Salzkotten, Germany; ac St. Vincenz Kliniken Salzkotten + Paderborn, Paderborn, Germany; ad Department of Gynecology and Obstetrics, Düsseldorf University Hospital, Düsseldorf, Germany; ae Center for Integrated Oncology Aachen Bonn Köln Düsseldorf, Düsseldorf, Germany; af Department of Medical Oncology, University Hospital Heidelberg, Germany; ag Division of Gynecologic Oncology, National Center for Tumor Diseases Heidelberg, Germany; ah German Cancer Consortium (DKTK) and German Cancer Research Center (DKFZ), Heidelberg, Germany

**Keywords:** Advanced breast cancer, Metastasis, de novo metastatic breast cancer, First line therapy, Hormone receptor-positive, HER2-negative

## Abstract

**Background:**

Patients with first-line metastatic breast cancer (MBC) comprise patients with de novo metastases (dnMBC) or recurrent disease after primary breast cancer (rMBC). This analysis aimed to explore the prognostic value of dnMBC versus rMBC overall and particularly in subgroups according to age and metastasis site, in addition to other prognostic clinicopathological parameters in a first-line, hormone receptor (HR)-positive, HER2-negative (HRpos/HER2neg) population.

**Methods:**

Within the prospective PRAEGNANT MBC registry (NCT02338167), 508 HRpos/HER2neg patients, receiving first-line treatment for advanced disease, were identified. Clinicopathological parameters (age, body mass index, performance status, tumor grading, metastasis site and therapy) were assessed according to metastatic status (dnMBC, rMBC within 5 years of primary diagnosis (rMBC <5 years), rMBC after more than 5 years (rMBC ≥5 years)). Cox regression analyses were performed to investigate whether metastatic status influences progression-free survival (PFS) and overall survival (OS).

**Results:**

*De novo* metastatic disease was present in 180 patients (35.4 %), whereas 132 patients (26.0 %) had rMBC <5 years and 196 patients (38.6 %) had rMBC ≥5 years. Patients with dnMBC had the most favorable prognosis. Relative to dnMBC, hazard ratios for PFS were 1.75 (95%CI: 1.31–2.34) in rMBC<5 years and 1.25 (95%CI: 0.94–1.65) for rMBC ≥5 years. Subgroup-specific differences were not observed.

**Conclusion:**

HRpos/HER2neg first-line MBC patients have a more favorable prognosis if the disease was previously not treated. This difference was similar across all examined clinicopathological parameters. It may therefore be beneficial to incorporate MBC categories as a stratification factor in clinical trials.

## Highlights

• Better prognosis with de novo than recurrent HRpos/HER2neg metastatic breast cancer.
• Clinicopathological parameter were similar in de novo and recurrent disease.
• Clinical trials may benefit from using metastasis categories as stratification factors.

## 1 Introduction

In the U.S. and Germany, 7 % of all primary breast cancer cases are diagnosed at stage IV (de novo metastatic breast cancer (dnMBC)) [1,2]. In contrast, approximately 30 % of primary cases without metastases will eventually develop recurrent metastatic breast cancer (rMBC) [1,2]. From the treatment perspective in the advanced breast cancer setting, this population constitutes the greater part of patients who commence therapy for metastatic breast cancer, with studies typically reporting figures between 50 % and 80 % [[3], [4], [5], [6], [7], [8]]. Importantly, rMBC patients already received prior loco-regional and systemic treatments for breast cancer. In contrast, dnMBC patients are treatment-naïve, thus presenting a group without secondary (acquired) resistances to any treatment. Nevertheless, it is possible that a certain percentage of patients with dnMBC have unidentified primary (intrinsic) resistances, which could affect prognosis.

Several studies have evaluated the difference in prognosis between patients with dnMBC and rMBC. In general, it could be shown that patients with dnMBC have a more favorable prognosis than most rMBC patients [3,[9], [10], [11], [12], [13]]. Nonetheless, one Asian study could not find a difference in prognosis between both patient groups [14]. Further examination based on the molecular subtypes of tumors revealed that this difference in prognosis was mostly seen in patients with HER2-positive and hormone receptor (HR)-positive/HER2-negative (HRpos/HER2neg), and less in triple-negative breast cancer [9,15]. Additionally, in patients with rMBC, the time period between primary diagnosis and the occurrence of MBC seemed to influence prognosis considerably [3,12,15].

This difference in prognosis is of special importance for clinical trials in the metastatic setting, where dnMBC patients account for a substantial proportion of included patients. Among first line patients included into the recent CDK4/6 inhibitor (CDK4/6i) trials (MONALEESA-2, MONALEESA-7, MONARCH 3, PALOMA-2), the proportion of dnMBC patients ranged from 34 % to 41 % [[16], [17], [18], [19]]. In contrast, the MONALEESA-3 trial reported dnMBC in 17–20 % of patients [20]. Here, the fact that this trial allowed second line treatment should be taken into account [20]. Despite the reported general difference in prognosis between dnMBC and rMBC, in the CDK4/6i trials, the overall benefit of CDK4/6i + endocrine therapy vs. endocrine monotherapy did not appear to differ greatly between patients with dnMBC and rMBC [[16], [17], [18], [19]].

From a clinical perspective, it would be helpful to further predict prognosis in patients with dnMBC and compare those groups with different groups of patients with rMBC. Hence, this study aimed to explore the prognostic value of dnMBC versus rMBC overall and particularly in subgroups according to age and metastasis site, in addition to other prognostic clinicopathological parameters and focused on patients with HRpos/HER2neg MBC.

## 2 Materials and methods

### 2.1 The PRAEGNANT research network

The PRAEGNANT study (Prospective Academic Translational Research Network for the Optimization of the Oncological Health Care Quality in the Adjuvant and Advanced/Metastatic Setting; NCT02338167 [21]) is an ongoing, prospective breast cancer registry with a documentation system similar to that of a clinical trial. Recruitment started in July 2014. The aims of PRAEGNANT are to assess treatment patterns and quality of life and to identify patients who may be eligible for clinical trials or specific targeted treatments [[21], [22], [23], [24]]. Patients can be included at any time point during the course of their disease. Follow-up assessments for the locally advanced, inoperable/metastatic setting are updated every 3 months until month 24 and thereafter every 6 months in case there is no progression or change of therapy within three months of observation. The study was approved by the relevant ethics committees (ethical approval number: 234/2014BO1: first approval on June 17, 2014, approval of Amendment 1 on June 11, 2015, approval of Amendment 2 on March 18, 2019; Ethics Committee of the Medical Faculty, University of Tübingen, Tübingen, Germany). All patients included in the present study provided informed consent.

### 2.2 Patients

**Fig. 1:**
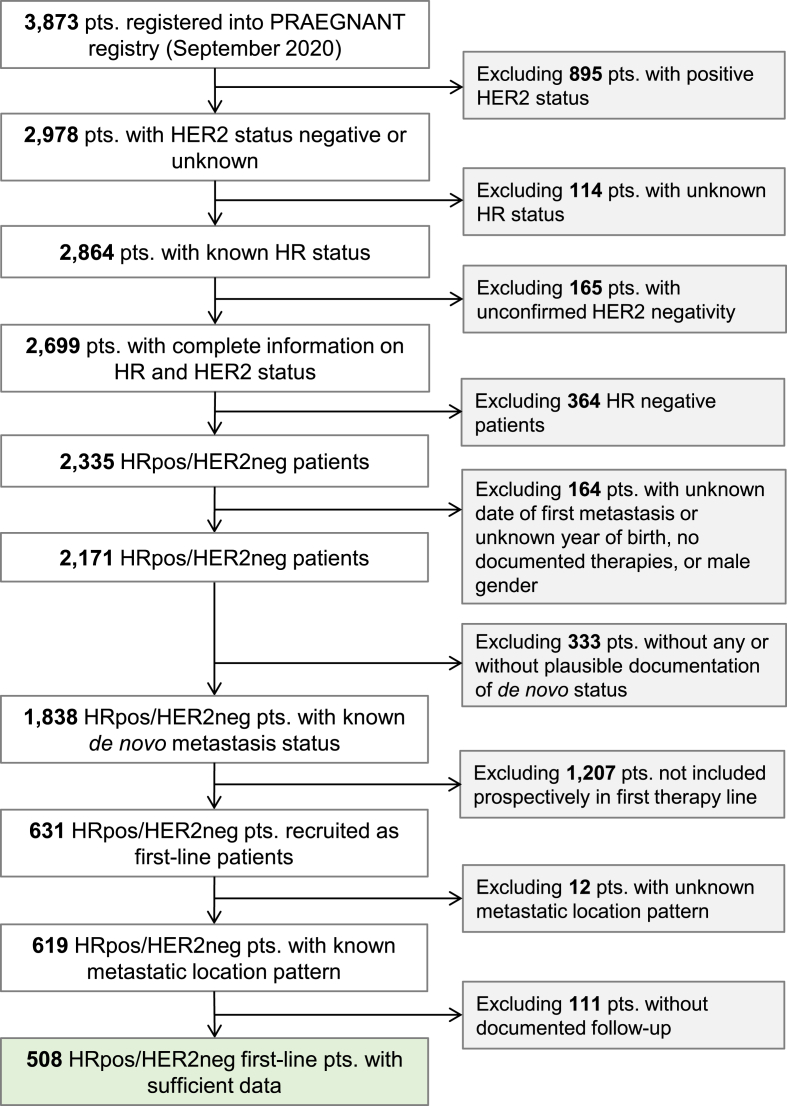
Patient flow chart. [HR: hormone receptor: pos: positive; neg: negative: pts.: patients].

At the time of database closure (September 26, 2020), 3873 patients were registered in the PRAEGNANT registry. Among them, 2171 had HRpos/HER2neg MBC. Patients were excluded if there was no (plausible) information on dnMBC status available (n = 333), if patients were not included prospectively during first line treatment (n = 1207), if the metastatic location pattern was unknown (n = 12) and if no follow-up was documented (n = 111). Therefore, the final patient population consisted of 508 patients. The patient flow chart is shown in Fig. 1.

### 2.3 Data collection

Data was collected by trained staff and documented in an electronic case report form [21]. Automated plausibility checks and on-site monitoring were performed. Data, generally not documented as part of routine clinical work, was collected prospectively using structured questionnaires completed on paper (epidemiological data such as family history, cancer risk factors, quality of life, nutrition and lifestyle items, and psychological health). Supplementary Table 1 provides an overview of the data collected.

### 2.4 Definition of HR status, HER2 status, and grading

The definition of HR status, HER2 status, and grading has been described previously [22]. In short, if a biomarker assessment of the metastatic site was available, this receptor status was used for the analysis. If there was no information for metastases, the latest biomarker results from the primary tumor were used. Additionally, all patients who received endocrine therapy in the metastatic setting were assumed to be HRpos, and all patients who had ever received anti-HER2 therapy were assumed to be HER2pos. There was no central review of biomarkers. The study protocol recommended assessing estrogen receptor and progesterone receptor status as positive if ≥ 1 % was stained. A positive HER2 status required an immunohistochemistry score of 3+ or positive fluorescence in situ hybridization/chromogenic in situ hybridization.

### 2.5 Statistical analysis

De novo metastases were defined as patients who were staged as cM1 during primary diagnosis (local diagnosis of breast cancer and consecutive disease staging), whereas rMBC was defined as being cM0 at primary diagnosis/staging. Patients with an implausible documentation of de novo state were excluded from analyses. In accordance with previous studies [7,11,12], a metastasis-free interval (MFI) of 92 days (∼3 months) was used. MFI was defined as the date of documented primary diagnosis until the date of documented metastases. dnMBC were expected to have an MFI <92 days and rMBC were expected to have an MFI >92 days. Correspondingly, patients, documented as cM1 at diagnosis/staging but with a documented MFI >92 days, and patients, documented as cM0 at diagnosis/staging but with a documented MFI <92 days were excluded from analyses due to having an implausible de novo status. (Fig. 1). rMBC was subdivided into rMBC with time from primary diagnosis to metastasis (TDTM) < 5 years and rMBC with TDTM ≥5 years. This cut-off was chosen to generate balanced and clinically relevant groups based on our sample size and the fact that later recurrences (>5 years) occur frequently in patients with HRpos/HER2neg metastatic breast cancer [7]. Progression-free survival (PFS) was defined as the time from first-line therapy begin to the earliest date of disease progression (distant-metastasis, local recurrence, or death from any cause) or the last date known to be progression-free. It was censored at 4 years, and it was left-truncated for time to enter the study if the entry was after therapy begin. Censoring was implemented to avoid imprecise estimation of survival rates due to the small number or patients still at risk at this time. Overall survival (OS) was defined in a similar fashion.

A multivariable Cox regression model for PFS (*basic model*) was fitted with the following predictors: age at diagnosis (continuous), body mass index (BMI, continuous), grading (categorical; G1/G2 vs. G3), ECOG (categorical; 0 vs. 1–4), metastasis site (categorical; brain, visceral, bone, other) and therapy group (categorical; CDK4/6 inhibitor, chemotherapy, other anti-hormone therapy). Subsequently, an additional Cox regression model (*full model*) was fitted containing the predictors of the basic model, metastasis status (categorical; dnMBC, rMBC and TDTM <5 years, rMBC and TDTM ≥5 years) and the interactions of metastasis status with age and metastasis site. The full model was compared to the basic model using a likelihood ratio test (LRT). In case of a significant result, the interaction model was compared with a reduced Cox regression model using the LRT: the basic model with metastasis status added but without the interaction terms (*reduced model*), using the LRT again. In case of a significance, adjusted subgroup-specific hazard ratios for metastasis status were calculated using the full model. Otherwise, adjusted overall hazard ratios for metastasis status were calculated, using the reduced model. A similar analysis had been planned for OS. As the number of events was too small to perform a statistical test with an interaction model with a reasonable test power, solely a reduced Cox regression model was compared to the basic model using an LRT and adjusted overall hazard ratios were estimated.

Missing values of predictors were imputed as done in Salmen et al. [25]. The proportional hazards assumptions were checked using the method of Grambsch and Therneau [26]. Survival rates were estimated using the Kaplan-Meier product limit method. As sensitivity analyses, unadjusted hazard ratios were estimated using a univariable Cox regression model for metastasis status solely. Survival rates were estimated using the Kaplan-Meier product limit method.

All of the tests were two-sided, and *P* < 0.05 was regarded as statistically significant. Analyses were carried out using R (version 3.6.1; R Development Core Team, Vienna, Austria, 2019).

## 3 Results

### 3.1 Clinicopathological parameters

All analyses were performed in the final population of 508 patients with HRpos/HER2neg advanced breast cancer who had not been treated previously for MBC. Baseline characteristics for the total population and grouped by metastasis status are shown in Table 1. A total of 180 patients (35.4 %) had dnMBC, 132 (26.0 %) had an rMBC within the first 5 years after primary diagnosis and 196 (38.6 %) developed an rMBC after more than 5 years. Patients were 54.1 ± 12.9 years old. Patients with rMBC were younger than those with dnMBC (52.0 ± 13.5 years for rMBC with TDTM <5 years; 50.6 ± 11.2 years rMBC with TDTM ≥5 years; 59.5 ± 12.5 years for dnMBC). In addition, patients who had a disease recurrence within the first 5 years more often presented with visceral metastases than the other groups (49.2 % in rMBC <5 years; 43.4 % in rMBC ≥5 years; 39.4 % in dnMBC). Patients with metastatic disease most frequently received first line treatment with a CDK4/6i (44.2 % of patients). Alternatively, 35.7 % of first line patients received chemotherapy. The percentage of chemotherapies was highest in patients who had rMBC with TDTM <5 years (48.1 % of patient receiving chemotherapy) and lowest in patients who had rMBC with TDTM ≥5 years (25.1 % of patients). Prior (neo)adjuvant chemotherapy was comparable between rMBC with TDTM <5 years and rMBC with TDTM ≥5 years, while a higher percentage of patients with rMBC with TDTM ≥5 years received (neo)adjuvant endocrine therapy than those with rMBC with TDTM <5 years (89.3 % vs. 77.6 %). Other clinicopathological parameters were comparable between groups (Table 1).

**Table 1:** Baseline characteristics, values are n (%) if not declared otherwise (dnMBC: de novo metastatic breast cancer; rMBC: recurrent metastatic breast cancer after primary breast cancer; TDTM: Time from diagnosis of primary breast cancer to metastasis; SD: standard deviation).

Characteristic		All patients (N = 508)	dnMBC (N = 180)	rMBC (N = 328)	
				TDTM <5 years (N = 132)	TDTM ≥5 years (N = 196)
Age at diagnosis (years)	mean (SD)	54.1 (12.9)	59.5 (12.5)	52.0 (13.5)	50.6 (11.2)
	missing	0	0	0	0
Body mass index (kg/m^2^)	mean (SD)	26.3 (5.6)	27.3 (6.5)	25.8 (5.2)	25.8 (4.8)
	missing	54	15	11	18
Grading	G1	33 (6.8)	14 (8.3)	7 (5.3)	12 (6.6)
	G2	303 (62.9)	103 (60.9)	70 (53.4)	130 (71.4)
	G3	146 (30.3)	52 (30.8)	54 (41.2)	40 (22.0)
	missing	26	11	1	14
Metastasis site	Brain	28 (5.5)	11 (6.1)	8 (6.1)	9 (4.6)
	Visceral	221 (43.5)	71 (39.4)	65 (49.2)	85 (43.4)
	Bone	151 (29.7)	59 (32.8)	36 (27.3)	56 (28.6)
	other	108 (21.3)	39 (21.7)	23 (17.4)	46 (23.5)
	missing	0	0	0	0
ECOG	0	270 (57.1)	95 (56.2)	79 (62.2)	96 (54.2)
	1	153 (32.3)	48 (28.4)	37 (29.1)	68 (38.4)
	2	30 (6.3)	12 (7.1)	9 (7.1)	9 (5.1)
	3	19 (4.0)	13 (7.7)	2 (1.6)	4 (2.3)
	4	1 (0.2)	1 (0.6)	0 (0.0)	0 (0.0)
	missing	35	11	5	19
Therapy	CDK4/6 inhibitor	223 (44.2)	75 (42.1)	50 (38.2)	98 (50.3)
	Chemotherapy	180 (35.7)	67 (37.6)	64 (48.9)	49 (25.1)
	Other	101 (20.0)	36 (20.2)	17 (13.0)	48 (24.6)
	Missing	4	2	1	1
(neo)adjuvant chemotherapy	Yes	–	–	98 (78.4)	133 (74.7)
	No	–	–	27 (21.6)	45 (25.3)
	missing	–	–	7	18
(neo)adjuvant endocrine therapy	Yes	–	–	97 (77.6)	159 (89.3)
	No	–	–	28 (22.4)	19 (10.7)
	missing	–	–	7	18

### 3.2 Progression-free survival

**Fig. 2:**
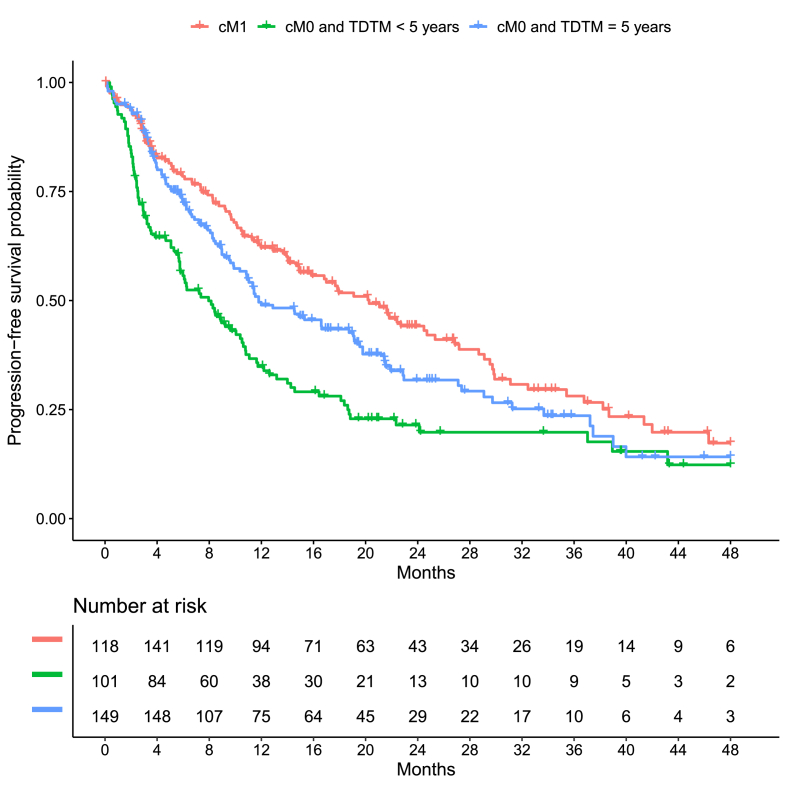
Kaplan-Meier curves for progression-free survival for status of metastasis at baseline combined with time from diagnosis to metastasis.

Median follow-up for PFS was 9.5 months. Cox regression analysis showed that metastasis status (dnMBC/rMBC TDTM <5 years/rMBC TDTM ≥5 years) influenced PFS (p < 0.01), irrespective of known prognostic predictors. Further analysis revealed that the effect of metastasis status on prognosis did not differ significantly between patient subgroups defined by age and metastasis site (p = 0.14). Relative to patients with dnMBC, patients with rMBC TDTM <5 years had an adjusted hazard ratio of 1.75 (95%CI: 1.31–2.34; p < 0.001) and patients with rMBC TDTM ≥5 years had a hazard ratio of 1.25 (95%CI: 0.94–1.65; p = 0.12). Unadjusted hazard ratios were similar and both adjusted and unadjusted hazard ratios are depicted in Table 2. Kaplan-Meier curves for PFS and metastasis status are shown in Fig. 2. Median PFS time for dnMBC, rMBC TDTM <5 years and rMBC ≥5 years were 20.3 months (95%CI: 14.9–26.8), 8 months (95%CI: 5.7–10.6) and 11.8 months (95%CI: 10.3–19.1) respectively. Median PFS times and survival rates are shown in Table 3.

**Table 2:** Hazard ratios and 95 % confidence intervals for metastasis status.

Outcome	Metastasis status	Adjusted analysis^a^		Unadjusted analysis	
		Hazard ratio (95%CI)	*P* Value	Hazard ratio (95 % CI)	*P* Value
**PFS**	**dnMBC**	Reference	–	Reference	–
	**rMBC TDTM < 5 years**	1.75 (1.31, 2.34)	<0.001	1.88 (1.42, 2.48)	<0.00001
	**rMBC TDTM ≥ 5 years**	1.25 (0.94, 1.65)	0.12	1.27 (0.97, 1.65)	0.08
**OS**	**dnMBC**	Reference	–	Reference	–
	**rMBC TDTM < 5 years**	2.04 (1.34, 3.10)	<0.001	2.12 (1.41, 3.18)	<0.001
	**rMBC TDTM ≥ 5 years**	1.42 (0.93, 2.17)	0.10	1.29 (0.86, 1.93)	0.21

*Abbreviations*: OS: overall survival: PFS: progression-free survival; 95%CI: 95 % confidence interval; dnMBC: de novo metastatic breast cancer; rMBC: recurrent metastatic breast cancer after primary breast cancer; TDTM: Time from diagnosis of primary breast cancer to metastasis.

a Hazard ratios are adjusted for age at diagnosis, body mass index, tumor grading, ECOG, therapy at first line and metastasis site.

**Table 3:** Survival rates and median survival times. (PFS: progression-free survival; OS: overall survival; dnMBC: de novo metastatic breast cancer; rMBC: recurrent metastatic breast cancer after primary breast cancer; TDTM: Time from diagnosis of primary breast cancer to metastasis).

Outcome	Group	Patients at risk			Events	Median survival time (months)	Survival rates		
		Total^a^	Max^b^	Start^c^			1-year	2-year	3-year
PFS	dnMBC	180	160	118	104	20.3 (14.9, 26.8)	0.62 (0.55, 0.70)	0.44 (0.37, 0.53)	0.28 (0.21, 0.38)
	rMBC TDTM <5 years	132	112	101	96	8.0 (5.7, 10.6)	0.35 (0.27, 0.45)	0.21 (0.15, 0.31)	0.20 (0.13, 0.30)
	rMBC TDTM ≥5 years	196	174	149	118	11.8 (10.3, 19.1)	0.49 (0.42, 0.57)	0.32 (0.25, 0.41)	0.24 (0.17, 0.33)
OS	dnMBC	180	166	118	44	–	0.89 (0.84, 0.94)	0.77 (0.71, 0.85)	0.75 (0.68, 0.83)
	rMBC TDTM <5 years	132	126	101	50	31.1 (25.1, NA)	0.80 (0.72, 0.87)	0.59 (0.50, 0.70)	0.45 (0.35, 0.59)
	rMBC TDTM ≥5 years	196	187	166	53	47.8 (32.1, NA)	0.87 (0.82, 0.93)	0.74 (0.67, 0.82)	0.51 (0.41, 0.63)

a Total number of patients with a follow-up period.

b The maximal number of patients who were simultaneously observed/at risk.

c The number of patients who were observed/at risk at time point 0. Some patients entered the study after therapy begin (time point 0) and were therefore not at risk from therapy begin to study entry.

### 3.3 Overall survival

**Fig. 3:**
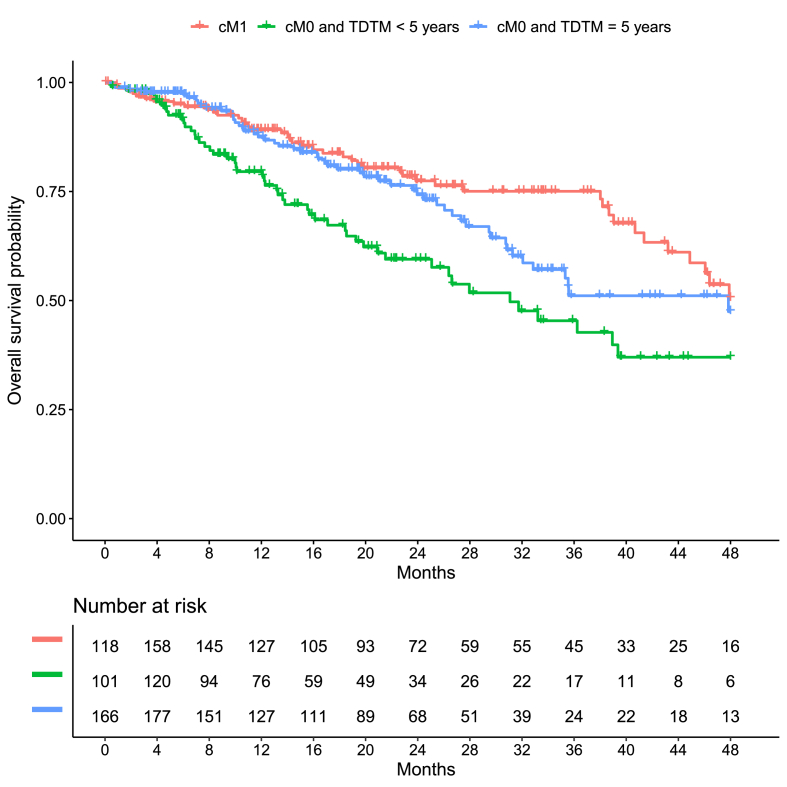
Kaplan-Meier curves for overall survival for status of metastasis at baseline combined with time from diagnosis to metastasis.

Median follow-up for OS was 18.0 months. Cox regression analyses showed that metastatic status (dnMBC/rMBC TDTM <5 years/rMBC TDTM ≥5 years) significantly affected OS (p < 0.01). Further subgroup-specific analyses could not be carried out due to small sample size. Adjusted and unadjusted HRs are shown in Table 2. Relative to dnMBC patients, patients with a rMBC TDTM <5 years and rMBC ≥ TDTM 5 years had hazard ratios of 2.04 (95%CI: 1.34–3.10; p < 0.001) and 1.42 (95%CI: 0.93–2.17; p = 0.10) respectively. Unadjusted hazard ratios were comparable. Kaplan-Meier curves for the OS are shown in Fig. 3 and median survival times and survival rates are shown in Table 3.

## 4 Discussion

In a population of HRpos/HER2neg breast cancer patients treated for first line metastatic disease, approximately one-third of patients had dnMBC, whereas two-thirds had rMBC. Patients with dnMBC had a better prognosis for both PFS and OS than rMBC patients. This difference was significant between dnMBC patients and rMBC patients who experienced recurrence within five years after primary diagnosis. Analyses for clinicopathological parameters could not show that this effect was different in certain groups.

In our patient HRpos/HER2neg patient population, 35.4 % of patients had dnMBC and 64.5 % had rMBC, which is slightly different than other studies in this cancer subtype, where the dnMBC to rMBC ratio was around 25/75 % [6,9]. Several studies have investigated the difference in prognosis between dnMBC and rMBC [3,4,9,[11], [12], [13], [14], [15],^27^]. In a Japanese study on patients with HRpos/HER2neg disease, dnMBC was associated with a more favorable OS (hazard ratio: 0.679, 95%CI: 0.429–1.075), although significance was not reached, which could be due to the limited sample size (65 patients with dnMBC vs. 107 patients with rMBC) [15]. Most other studies, with the exception of one other Japanese study ([14]), also observed a prognostic benefit for patients with dnMBC. Some of these studies presented subgroup analyses according to molecular subtype or year of diagnosis and could show that the prognostic difference between dnMBC and rMBC is most prominent in patients with HER2pos and HRpos/HER2neg breast cancer, and to a lesser extent in triple negative breast cancer [4,8,9,11,13,28]. In addition, the prognosis of dnMBC has shown improvements over the last decades [4]. A study that evaluated the characteristics of patients with hormone receptor-positive breast cancer who progressed within or after 5 years found that patients with a distant recurrence-free interval (DRFI) < 5 years had higher-grade tumors and were more often treated with chemotherapy [29], which is in line with our results. Several studies have noted that patients with rMBC who have longer metastasis-free interval have a better prognosis than patients who metastasize earlier ([3,9,12,29]. Here, we could also only observe a significant prognostic benefit for dnMBC versus rMBC that progressed within 5 years, but not versus rMBC that progressed after 5 years.

Our analysis with interaction terms between dnMBC status and other clinicopathological parameters did not indicate that the effect of dnMBC on prognosis is different in certain subgroups. Nevertheless, dnMBC patients less often had visceral metastases than patients with rMBC, which has also been observed in other studies [4,12,30]. Due to the limited sample size, only few interactions could be analysed, and the power might have been too low to obtain significant results. Some additional clinicopathological characteristics that have been evaluated in other studies and have shown to be different between dnMBC and rMBC include tumor size, nodal involvement and histological type of the primary tumor [4,30].

The observed differences in prognosis between dnMBC and rMBC patients are important in the context of clinical studies. Although the patient population of the recent CDK4/6i trials in the first line setting had a substantial amount of dnMBC patients, there was no systematic difference in benefit of CDK4/6i over anti-hormone monotherapy between dnMBC and rMBC patients [[16], [17], [18], [19]]. Also, the fact that some of these studies only included postmenopausal women, while other also included pre- and perimenopausal women, could indicate that the effect of dnMBC might not be different in some subgroups of the patient population. Nevertheless, additional research is still needed.

Our study has some limitations. First, our patient population is relatively young (on average 54.1 years old), which could have affected analyses. The process of providing informed consent in our registry may have influenced patient enrollment, as some patient groups may be more or less inclined to provide informed consent. This may thus be related to the younger age of our patient population. Second, the proportion of patients who were treated with first line chemotherapy is relatively high (35.7 % of the patients). Notably, a large percentage of patients were recruited into the PRAEGNANT registry before CDK4/6i availability. A previous publication showed that before the introduction of CDK4/6i, approximately 42 % of the first line MBC patients were treated with chemotherapy [22]. Furthermore, recent analyses in our registry showed that the percentage of patients receiving first line chemotherapy has continuously decreased since CDK4/6i introduction [31,32]. The examined patient population might thus not accurately represent the first line MBC population that is being treated today. Furthermore, the therapy landscape might change even more in the coming year due to the development of new therapies [33]. Notwithstanding, the inclusion of a substantial number of patients receiving chemotherapy facilitated the examination of the effect of metastatic status according to therapy type. Third, in the current analysis, sample size restrictions precluded dividing rMBC patients into more than two subgroups. Although the cut-off of 5 years generated clinically relevant and balanced groups, with this cut-off also being used in other studies, there might be subgroups within the TDTM >5 years group that may have a better prognosis than others, which we were not able to evaluate here. Indeed, disease recurrence in patients with HRpos/HER2neg breast cancer can extend beyond 10 years and these patients that eventually progress later may have a better prognosis than those progressing earlier.

## 5 Conclusions

Patients with HRpos/HER2neg MBC who are treated with first line therapy have a more favorable prognosis if the disease was previously not treated (dnMBC) compared to patients who had a recurrence of the disease (rMBC). Specific clinicopathological factors that explained this observed prognostic difference could not be identified. Although the inclusion of patients from both populations into first-line MBC trials seems feasible, it may be necessary to incorporate MBC categories as a stratification factor in clinical trials. Nevertheless, more research is needed to evaluate the effect of MBC-categories on treatment effectiveness.

## CRediT authorship contribution statement

**Christina B. Walter:** Writing – review & editing, Investigation, Conceptualization. **Andreas D. Hartkopf:** Writing – review & editing, Supervision, Investigation, Conceptualization. **Alexander Hein:** Writing – review & editing, Investigation. **Peter A. Fasching:** Writing – original draft, Supervision, Methodology, Investigation, Funding acquisition, Data curation. **Hans-Christian Kolberg:** Writing – review & editing, Investigation. **Peyman Hadji:** Writing – review & editing, Investigation. **Hans Tesch:** Writing – review & editing, Investigation. **Lothar Häberle:** Writing – review & editing, Methodology, Formal analysis. **Johannes Ettl:** Writing – review & editing, Investigation. **Diana Lüftner:** Writing – review & editing, Investigation. **Markus Wallwiener:** Writing – review & editing, Investigation. **Volkmar Müller:** Writing – review & editing, Investigation. **Matthias W. Beckmann:** Writing – review & editing, Investigation. **Laura L. Michel:** Writing – review & editing, Investigation. **Erik Belleville:** Writing – review & editing, Project administration, Funding acquisition. **Hanna Huebner:** Writing – original draft, Funding acquisition. **Sabrina Uhrig:** Writing – review & editing, Data curation. **Chloë Goossens:** Writing – review & editing, Writing – original draft, Investigation. **Pauline Wimberger:** Writing – review & editing, Investigation. **Carsten Hielscher:** Writing – review & editing, Investigation. **Julia Meyer:** Writing – review & editing, Methodology, Formal analysis. **Christoph Mundhenke:** Writing – review & editing, Investigation. **Christian Kurbacher:** Writing – review & editing, Investigation. **Rachel Wuerstlein:** Writing – review & editing, Investigation. **Michael Untch:** Writing – review & editing, Investigation. **Wolfgang Janni:** Writing – review & editing, Investigation. **Florin-Andrei Taran:** Writing – review & editing, Investigation. **Michael P. Lux:** Writing – review & editing, Investigation. **Diethelm Wallwiener:** Writing – review & editing, Investigation. **Sara Y. Brucker:** Writing – review & editing, Investigation. **Andreas Schneeweiss:** Writing – review & editing, Investigation. **Tanja N. Fehm:** Writing – review & editing, Investigation. **Carlo Fremd:** Writing – review & editing, Investigation, Conceptualization.

## Declaration of competing interest

**CBW:** has received honoraria from Teva, AstraZeneca, Novartis, Pfizer, and Roche. **ADH**: has received honoraria from Roche, Novartis, Lilly, MSD, AstraZeneca, Seagen, GSK, Exact Science, Riemser, Teva, Onkowissen, Gilead, Stemline Therapeutics, Pfizer, Amgen, Pierre Fabre and Eisai and travel support from Roche, Novartis, Lilly, AstraZeneca, GSK, Exact Science, Gilead, Stemline Therapeutics and Pfizer. **PAF**: has received honoraria from Roche, Pfizer, Novartis, and Celgene; his institution conducts research for Novartis. **H-CK:** has received honoraria from Pfizer, Novartis, Roche, Genomic Health/Exact Sciences, Amgen, AstraZeneca, Riemser, Carl Zeiss Meditec, TEVA, Theraclion, Janssen-Cilag, GSK, LIV Pharma, Lily, SurgVision, Onkowissen, Gilead, Daiichi Sankyo and MSD, travel support from Carl Zeiss Meditec, LIV Pharma, Novartis, Amgen, Pfizer, Daiichi Sankyo, Tesaro, Gilead and Stemline Therapeutics and owns stock of Theraclion SA. **PH**: has received honoraria, unrestricted educational grants, and research funding from Amgen, Novartis, Hexal and Pfizer. **HT**: has received honoraria from Novartis, Roche, Celgene, Teva, and Pfizer, as well as travel support from Roche, Celgene, and Pfizer. **JE** has received honoraria/travel support from Roche, Celgene, Novartis, Pfizer, Lilly, Pierre Fabre, Teva, and Tesaro, AstraZeneca, Daiichi Sankyo, Seagen, Gilead, Stemline Therapeutics and ClinSol. **DL:** has received honoraria from Amgen, Loreal, Pfizer, Novartis, Eli Lilly, Samsung, Celgene, Astra Zeneca, Teva and GSK. **MW**: has received speaker honoraria from AstraZeneca, Celgene, Roche, MSD and Novartis. **VM**: has received speaker honoraria from AstraZeneca, Daiichi Sankyo, Eisai, Pfizer, MSD, Medac, Novartis, Roche, Seagen, Onkowissen, high5 Oncology, Medscape, Gilead, Pierre Fabre, iMED Institut, has received consultancy honoraria from Roche, Pierre Fabre, PINK, ClinSol, Novartis, MSD, Daiichi Sankyo, Eisai, Lilly, Seagen, Gilead, Stemline. Has received institutional research support from Novartis, Roche, Seagen, Genentech, AstraZeneca. Has received travel grants from AstraZeneca, Roche, Pfizer, Daiichi Sankyo, Gilead. **LLM:** received honoraria from Amgen, AstraZeneca, Celgene, Gilead, Lilly, MSD, Novartis, Pfizer, Roche and Eisai for advisory boards, lectures and travel support. **EB**: has received honoraria from Novartis, Celgene, Eisai, Daiichi Sankyo, Merrimack, AstraZeneca, Riemser, Pfizer, Hexal, Amgen, and onkowissen.de for consulting, clinical research management, or medical education activities. **HH**: received speaker honoraria from Novartis Pharma GmbH and LEO Pharma GmbH and Grant/Research support from Novartis Pharma GmbH. **PW**: has received honoraria from Roche, Novartis, Amgen, AstraZeneca, Pfizer, MSD, Clovis, Tesaro, Celgene, Teva, Eisai, Seagen, Daiichi Sankyo and Eli Lilly. **CH**: has received honoraria from Amgen, Celgene, Oncovis, Roche, and Pfizer. **CK**: has received honoraria from Amgen, Roche, Teva, Novartis, MSD, Axios, and Riemser. **RW**: has received honoraria from Agendia, Amgen, APOGHEVA, Aristo, Astra Zeneca, Celgene, Clovis Oncology, Daiichi Sankyo, Eisai, Esteve, Exact Sciences, Gilead, Glaxo Smith Kline, Hexal, Lilly, Medstrom Medical, MSD, Mundipharma, Mylan, Nanostring, Novartis, Odonate, Paxman, Palleos, Pfizer, Pierre Fabre, PINK, Puma Biotechnolgogy, Riemser, Roche, Sandoz/Hexal, Sanofi Genzyme, Seattle Genetics/Seagen, Sidekick, Stemline Therapeutics, Tesaro Bio, Teva, Veracyte, Viatris, Wiley, FOMF, Aurikamed, Clinsol, Pomme Med, medconcept, MCI, MediSeminar. **MU:** has received honoraria for advisory boards and travel support, payed to the employer from Abbvie, Amgen, AstraZeneca, BMS, Celgene, Daiichi Sankyo, Eisai, Lilly Deutschland, Lilly Int., MSD, Mundipharma, Myriad Genetics, Odonate, Pfizer, Puma Biotechnology, Roche, Sanofi Aventis Deutschland, Teva Pharmaceuticals Ind Ltd, Novartis, Pierre Fabre, Clovis Oncology, and Seattle Genetics. **WJ:** as received honoraria and research grants from Sanofi-Aventis, Novartis, Lilly, Pfizer, Roche, Chugai, AstraZeneca, MSD, and Daiichi Sankyo. **F-AT**: has received speaker and consultancy honoraria from Astra Zeneca, Gilead, GSK, MSD, Novartis, Onkowissen, Pfizer, Roche. **MPL**: has received honoraria from Lilly, Pfizer, Roche, MSD, Hexal, Novartis, AstraZeneca, Eisai, Exact Sciences, Agendia, Daiichi Sankyo, Grünenthal, Gilead, Pierre Fabre, PharmaMar, Samantree, Endomag, and medac for advisory boards, lectures, and travel support. **SYB:** has received honoraria from Roche, Novartis, Pfizer, MSD, Teva, and AstraZeneca. **AS**: has received honoraria from Roche, Celgene, AstraZeneca, Novartis, Pfizer, Zuckschwerdt Verlag GmbH, Georg Thieme Verlag, Aurikamed GmbH, MCI Deutschland GmbH, bsh medical communications GmbH, and promedicis GmbH. **TNF:** has received honoraria from Novartis, Roche, Pfizer, Teva, Daiichi Sankyo, AstraZeneca, and MSD. **CF** has received honoraria from Pfizer, Roche, Eisai, AstraZeneca, Lilly, MSD, Novartis, Gilead and Veracyte. All other authors declare no conflict of interest.
