# Supplementary material for: Characteristics and prognosis of patients with primary metastatic disease vs. recurrent HER2-negative, hormone receptor-positive advanced breast cancer
Source: Breast. 2025 Feb 5;80:104412. doi: 10.1016/j.breast.2025.104412 (PMC11872390; doi:10.1016/j.breast.2025.104412)
Supplement: Multimedia component 1 [file mmc1.docx]

Supplementary material for:

Characteristics and prognosis of patients with primary metastatic disease vs. recurrent HER2-negative, hormone receptor-positive advanced breast cancer

**Christina B. Walter ^1^, Andreas D. Hartkopf ^1^, Alexander Hein ^2^, Peter A. Fasching ^2^, Hans-Christian Kolberg ^3^, Peyman Hadji ^4^, Hans Tesch ^5^, Lothar Häberle ^2,6^, Johannes Ettl ^7,8^, Diana Lüftner ^9^, Markus Wallwiener ^10^, Volkmar Müller ^11^, Matthias W. Beckmann ^2,12^, Laura L. Michel ^13^, Erik Belleville ^14^, Hanna Huebner ^2^, Sabrina Uhrig ^2^, Chloë Goossens ^2^, Pauline Wimberger ^15,16,17^, Carsten Hielscher ^18^, Julia Meyer ^2,6^, Christoph Mundhenke ^19^, Christian Kurbacher ^20^, Rachel Wuerstlein ^21^, Michael Untch ^22^, Wolfgang Janni ^23^, Florin-Andrei Taran ^24^, Michael P. Lux ^25^, Diethelm Wallwiener ^1^, Sara Y. Brucker^1^, Andreas Schneeweiss ^13^, Tanja N. Fehm ^26,27^, Carlo Fremd ^28,29,30^**

1. Department of Obstetrics and Gynecology, University of Tübingen, Tübingen, Germany
2. Department of Gynecology and Obstetrics, Erlangen University Hospital, Comprehensive Cancer Center Erlangen-EMN, Friedrich Alexander University of Erlangen–Nuremberg, Germany
3. Department of Gynecology and Obstetrics, Marienhospital Bottrop, Bottrop, Germany
4. Frankfurt Center for Bone Health, Frankfurt am Main, Germany
5. Oncology Practice, Bethanien Hospital, Frankfurt am Main, Germany
6. Biostatistics Unit, Department of Gynecology and Obstetrics, Erlangen University Hospital, Erlangen, Germany
7. Department of Obstetrics and Gynecology, Klinikum rechts der Isar, Technical University of Munich, Munich, Germany
8. Cancer Center Kempten/Allgäu (CCKA), Klinikum Kempten, Kempten, Germany
9. Immanuel Hospital Märkische Schweiz & Immanuel Campus Rüdersdorf, Medical University of Brandenburg Theodor-Fontane, Rüdersdorf bei Berlin, Germany
10. Department of Gynecology, Halle University Hospital, Halle, Germany
11. Department of Gynecology, Hamburg-Eppendorf University Medical Center, Hamburg, Germany
12. Bavarian Center for Cancer Research (BZKF), Erlangen, Germany.
13. National Center for Tumor Diseases, Heidelberg University Hospital, German Cancer Research Center (DKFZ), Heidelberg, Germany
14. ClinSol GmbH & Co KG, Würzburg, Germany
15. Department of Gynecology and Obstetrics, Carl Gustav Carus Faculty of Medicine and University Hospital, TU Dresden, Dresden, Germany
16. National Center for Tumor Diseases (NCT), Dresden, Germany: German Cancer Research Center (DKFZ), Heidelberg, Germany; Carl Gustav Carus Faculty of Medicine and University Hospital, Technical University of Dresden, Dresden, Germany; Helmholtz-Zentrum Dresden-Rossendorf (HZDR), Dresden, Germany
17. German Cancer Consortium (DKTK), Dresden and German Cancer Research Center (DKFZ), Heidelberg, Germany
18. g.SUND Gynäkologie-Onkologisches Zentrum, Stralsund, Germany
19. Department of Gynecology and Obstetrics, Klinik Hohe Warte, Bayreuth, Germany
20. Department of Gynecology I (Gynecologic Oncology), Gynecologic Center Bonn-Friedensplatz, Bonn, Germany
21. Breast Center and CCC Munich, Deptartment of Gynecology and Obstetrics, University Hospital LMU Munich, Munich, Germany
22. Department of Gynecology and Obstetrics, Helios Clinics Berlin-Buch, Berlin, Germany
23. Department of Gynecology and Obstetrics, Ulm University Hospital, Ulm, Germany
24. Department of Obstetrics and Gynecology, University Medical Center Freiburg, Freiburg, Germany
25. Department of Gynecology and Obstetrics, Frauenklinik St. Louise, Paderborn, St. Josefs-Krankenhaus, Salzkotten, Germany; St. Vincenz Kliniken Salzkotten + Paderborn, Paderborn, Germany
26. Department of Gynecology and Obstetrics, Düsseldorf University Hospital, Düsseldorf, Germany
27. Center for integrated oncology Aachen Bonn Köln Düsseldorf, Düsseldorf, Germany
28. Department of Medical Oncology, University Hospital Heidelberg, Germany
29. Division of Gynecologic Oncology, National Center for Tumor Diseases Heidelberg, Germany
30. German Cancer Consortium (DKTK) and German Cancer Research Center (DKFZ), Heidelberg, Germany

**Supplementary Table 1.**Data categories recorded in the PRAEGNANT study.

| Data continuously captured, if applicable | Data assessed at study entry | Data assessed at follow-up care appointments |
| --- | --- | --- |
| Concomitant diseases | Life status, ECOG | Life status, ECOG |
| Concomitant medication | Quality of life | Quality of life |
| Cancer systemic therapies | Breast cancer risk factor questionnaire | Breast and axilla evaluation |
| Cancer radiotherapy | Breast and axilla evaluation | Distant metastasis evaluation |
| Cancer surgery | Distant metastasis evaluation | Biomaterial ascertainment |
| Breast cancer, right side | Biomaterial ascertainment | PRO questionnaires |
| Breast cancer, left side | PRO questionnaires |  |

ECOG, Eastern Cooperative Oncology Group (performance status); PRO, patient-reported outcome.
